# Extracts from *Aralia elata (Miq) Seem* alleviate hepatosteatosis via improving hepatic insulin sensitivity

**DOI:** 10.1186/s12906-015-0871-5

**Published:** 2015-10-05

**Authors:** Kyung-A Hwang, Yu-Jin Hwang, Ga Ram Kim, Jeong-Sook Choe

**Affiliations:** Department of Agrofood Resources, National Academy of Agricultural Science, RDA, Wanju-Gun, Jeollabuk-do 565-851 Republic of Korea

**Keywords:** *Aralia elata* (Miq) *Seem*, Non-Alcoholic Fatty Liver Disease, Insulin Resistance

## Abstract

**Background:**

Non-alcoholic fatty liver disease (NAFLD) is a common liver disease that is strongly associated with obesity and dysregulation of insulin in the liver. However, currently no pharmacological agents have been established for the treatment of NAFLD. In this regard, we sought to evaluate the anti-NAFLD effects of *Aralia elata* (Miq) *Seem* (AE) extract and its ability to inhibit hepatic lipid accumulation and modulate cellular signaling in a high fat diet (HFD)-induced obese mouse model.

**Methods:**

A model of hepatic steatosis in the HepG2 cells was induced by oleic acid. Intracellular lipid droplets were detected by Oil-Red-O staining, and the expression of sterol regulatory element-binding protein 1(SREBP-1), Fatty acid synthase (FAS), Acetyl-CoA carboxylase (ACC) 1 and 2, Peroxisome proliferator activated receptor-α (PPARα), and carnitine palmitoyl transferase 1(CPT-1) was analyzed by real time reverse transcription–Polymerase chain reaction (qRT–PCR). And glucose consumption was measured with commercial kit. Furthermore, Male C57BL/6 J mice were fed with HFD to induce NAFLD. Groups of mice were given plant extracts orally at 100 and 300 mg/kg at daily for 4 weeks. After 3 weeks of AE extract treatment, we performed oral glucose tolerance test (OGTT). Liver tissue was procured for histological examination, Phosphoinositide 3-kinase (PI3K) and Protein kinase B (PKB/Akt) activity.

**Results:**

In the present study, AE extract was shown to reduce hepatic lipid accumulation and significantly downregulate the level of lipogenic genes and upregulate the expression of lipolysis genes in HepG2 cells. And also, AE extract significantly increased the glucose consumption, indicating that AE extract improved insulin resistance. Subsequently, we confirmed the inhibitory activity of AE extract on NAFLD, in vivo. Treatment with AE extract significantly decreased body weight and the fasting glucose level, alleviated hyperinsulinism and hyperlipidemia, and reduced glucose levels, as determined by OGTT. Additionally, AE extract decreased PI3K and Akt activity.

**Conclusions:**

Our results suggest that treatment with AE extract ameliorated NAFLD by inhibiting insulin resistance through activation of the Akt/GLUT4 pathway.

**Electronic supplementary material:**

The online version of this article (doi:10.1186/s12906-015-0871-5) contains supplementary material, which is available to authorized users.

## Background

Excessive intake of dietary lipids can lead to fatty liver disease and to the development of liver lesions. These abnormalities can later progress to disease conditions such as non-alcoholic fatty liver disease (NAFLD) and non-alcoholic steatohepatitis (NASH) [1–3]. NAFLD is one of the most likely causes of abnormal liver function and is characterized by hepatic steatosis [4]. In 20 % of NAFLD, hepatic steatosis can progress to NASH, with some people ultimately developing cirrhosis and liver failure [5]. Although the etiology of NAFLD has not yet been clarified, it is epidemiologically strongly associated with obesity and dysregulated insulin activity in the liver [6, 7]. The principal function of insulin in the liver is to suppress glucose production when the blood glucose concentration increases abnormally. This process is impaired in hepatic insulin resistance (IR) and contributes to postprandial hyperglycemia. The development of hepatic IR is very closely linked to NAFLD [8] and based on these findings; studies of NAFLD treatment are mostly focused on reducing IR. A pharmacological approach that targets IR has more promising therapeutic effects than do lipid-lowering agents and anti-obesity drugs [9–13]. However, these medications have no significant effect or lack long-term safety and efficacy. Therefore, recently there has been a great interest in using the bioactive compounds derived from plants for treatment of NAFLD because of their low toxicity and fewer side effects [14–20].

*Aralia elata* (Miq) *Seem* (AE) is a shrub that belongs to the Araliaceae family and is widely distributed in oriental countries such as Korea, Japan, and China [21]. The young shoots of AE are a popular edible part of the plant, especially in the spring. Its barks and root cortexes are widely used in folk medicine for the treatment of diabetes, gastric ulcer, hepatitis, rheumatoid arthritis, and other cytotoxic and inflammatory conditions [22–25]. Recent studies have observed that AE extracts possess anti-diabetes and anti-obesity activities [26, 27]. An ethanol extract of AE was found to be effective in improving hyperglycemia and preventing diabetes [26]. In addition to this, a saponin extract from the shoot of AE significantly reduced serum glucose and cholesterol levels [27]. However, whether IR is targeted by the mechanism of action of AE extract has rarely been reported. Therefore, in the present study, we examined the beneficial effects of AE extract on hepatic fat accumulation and IR, confirming a relationship between reduced insulin resistance and NAFLD in AE extract treated-group.

## Methods

### Samples, antibodies, and reagents

AE extract, obtained by using 70 % ethanol, was purchased from the Plant Extract Bank (Jeju, Korea). Dulbecco’s modified Eagle’s medium (DMEM), fetal bovine serum (FBS), and penicillin–streptomycin (PS) were obtained from Gibco (Carlsbad, CA, USA). Oil-red-O and oleic acid (OA) were obtained from Sigma–Aldrich (Saint Louis, MO, USA). Cell Titer-Glo was obtained from Promega (Madison WI, USA). All other chemicals were purchased from Sigma–Aldrich unless specified otherwise.

### OA/BSA complex solution preparation

OA/BSA complex solution was prepared by a slight modification of previously described methods [28]. One hundred mM OA stock solution was prepared in 0.1 N NaOH by heating at 70 °C in a shaking water bath. In an adjacent water bath at 55 °C, a 10 % (w/v) FFA-free BSA solution was prepared in H_2_O. Twenty mM OA containing 10 % BSA was diluted in the culture medium to obtain the desired final concentrations. The OA/BSA complex solution was sterile-filtered through a 0.45 μm pore membrane filter and stored at −20 °C.

### Cell culture

The human hepatocellular carcinoma cell line HepG2 was obtained from the Korean Cell Line Bank (Seoul, Korea). HepG2 cells were cultured in DMEM supplemented with 10 % FBS and 1 % PS in an incubator with 5 % CO_2_ at 37 °C. To accumulate fatty acids, HepG2 cells were exposed to OA at a final concentration of 2 mM for 24 h.

### Cell viability

HepG2 cells seeded (1 × 10^5^ cells/well) in 24-well plates were treated with AE. AE ethanol extract in dimethyl sulfoxide (DMSO) was diluted with phosphate-buffered saline (PBS) to obtain final concentrations of 100, 200, and 500 μg/mL. Cells were treated with extract samples for 24 h, and cell viability measured with Cell Titer-Glo® (Promega). Viability is expressed as the percentage of live cells in each well.

### Staining using oil-red-O

HepG2 cells (2 × 10^5^ cells/mL) were treated with AE (100 μg/mL) and OA (2 mM) for 24 h. After incubation, cells were fixed with 4 % paraformaldehyde and stained with a freshly prepared working solution of Oil-red-O for 20 min at room temperature. After several washes, stained cells were observed under a microscope (Nikon, Tokyo, Japan).

To quantify Oil-red-O content, isopropanol was added to each sample. The sample was shaken at room temperature for 5 min, and the optical density of the isopropanol-extracted sample was determined using a spectrophotometer at 510 nm.

### Real-time Reverse Transcription–Polymerase Chain Reaction (RT–PCR) analyses

RT-PCR was used to quantify the expression of lipids. This was done using a Rotor-Gene Q Real-time Thermal Cycler (Qiagen, Stanford, VA, USA) according to the manufacturer’s instructions. HepG2 cells were treated with AE (100 μg/mL) and OA (2 mM) for 24 h. After incubation, total RNA isolated using RNeasy mini plus kit (Qiagen). In case of GLUT4 expression, the skeletal muscle was quickly freeze-clamped in situ and kept in liquid nitrogen until analyzed. Muscles were ground and mixed with lysis buffer. Homogenates were spun at 15,000 × g for 10 min at 4 °C, and total RNA isolated using RNeasy mini plus kit (Qiagen). cDNA was synthesized from the total RNA. The PCR was carried out using 2X SYBR Green mix (Qiagen). All results were normalized to the expression of glyceraldehydes-3-phosphate dehydrogenase (GAPDH). Primer sequences are given in Table 1.

**Table 1:** Gene-specific primers used for real-time RT–PCR

Gene	Forward	Reverse	Species
SREBP-1	5′-GCGGAGCCATGGATTGCAC-3′	5′-TCTTCCTTGATACCAGGCCC-3′	Homo sapiens
FAS	5′-AGCTGCCAGAGTCGGAGAAC-3′	5′-TGTAGCCCACGAGTGTCTCG-3′	
ACC1	5′-GAGGGCTAGGTCTTTCTGGAAG-3′	5′-CCACAGTGAAATCTCGTTGAGA-3′	
PPARα	5′-TCCGACTCCGTCTTCTTGAT-3′	5′-GCCTAAGGAAACCGTTCTGTG-3′	
CPT1	5′-TGAGCGACTGGTGGGAGGAG-3′	5′-GAGCCAGACCTTGAAGTAGCG-3′	
ACC2	5′-GCCAGAAGCCCCCAAGAAAC-3′	5′-CGACATGCTCGGCCTCATAG-3′	
GAPDH	5′-CGGAGTCAACGGATTTGGTCGTAT-3′	5′-AGCCTTCTCCATGGTGGTGAAGAC-3′	
GLUT4	5′-CAGCCTCTTCTCCTTCCTGAT-3′	5′-GCCAGAGGGCTGATTAGAGA-3′	Mus muscularis
GAPDH	5′-CGGAGTCAACGGATTTGGTCGTAT-3′	5′-AGCCTTCTCCATGGTGGTGAAGAC-3′	

### Glucose consumption assay

HepG2 cells seeded (2 × 10^5^ cells/mL) in 6-well plates and treated with OA (2 mM) for 24 h. After incubation, cells were incubated in glucose- and serum-free DMEM for another 2 h. And AE (100 μg/mL) and 7 mM glucose were treated in HepG2 cells for 24 h. The supernatant of each group were collected and the glucose level was measured using a glucose assay kit (Abcam, Cambridge, UK,).

### Animals

C57BL/6 mice were kept in a humidity-controlled room under a 12-h light–dark cycle, with food and water available ad libitum for 1 week. The mice were then divided randomly into five groups with five animals each. The 1 group of C57BL/6 mice was fed the standard rodent chow (Harlan Teklad Mouse/Rat Diet 7002). The other groups were fed a high-fat diet (HFD) that contained 60 % fat, 14 % protein, and 26 % carbohydrate. The 2 groups of the mice were administrated AE extracts by oral gavage, with 100 and 300 mg/kg, respectively. The well-known pharmaceutical drug for NAFLD, resveratrol (RV), was used as the positive control (at a dose of 300 mg/kg). The other 1 group was given equal volume of distilled water.

The study was approved by the Institutional Animal Care and Use Committee of the National Academy of Agricultural Science (NAAS-201411) and all procedures were conducted in accordance with Animal Experiments Guidelines of the National Academy of Agricultural Science.

### Basal study

At the end of a 4-week period, after overnight fasting, each animal was weighed, and blood samples were collected. The plasma was placed into aliquots for the respective analyses. Kits for determining plasma glucose concentration was purchased from Abcam. Commercial enzyme-linked immunosorbent assay (ELISA) kits were used to quantify plasma insulin concentration (Millipore, St. Charles, MO). Kits for determination of plasma levels of total triglycerides (TG) was purchased from Cayman Chemical Company. All experimental assays were carried out according to the manufacturer’s instruction. All samples were analyzed in triplicate. Whole-body insulin sensitivity was estimated using the homeostasis model assessment of insulin resistance (HOMA-IR) with the following formula: [fasting plasma glucose (mmol) × fasting plasma insulin (mU/ml)]/22.5 [29].

### Oral glucose tolerance test

On the 21th day following AE extract treatment, an oral glucose tolerance test (OGTT) was performed on all animals. OGTT was conducted using 2 g/kg of glucose. Blood samples were collected from the tail vein to measure glucose at 0, 30, 60, 90 120, and 240 min after glucose administration (po). The blood glucose levels were determined by a glucose meter (Roche, ACCU-CHEK Active).

### Histological analysis of the liver

The liver of each animal was fixed in 4 % buffered neutral formalin, embedded in paraffin, and cut into sections with a thickness of 4 μm. The sections were stained with hematoxylin and eosin (H&E) to evaluate the degree of hepatic steatosis. All slides were scanned at a total magnification of 200× using a microscope.

### Phosphoinositide 3- kinase and protein kinase B activity

Phosphoinositide (PI) 3- kinase (PI3K) kinase activity was performed using PI3K assay kit from Millipore. And protein kinase B (PKB/Akt) activity was performed according to methods described previously [30]. The primary antibody used for experiments was rabbit polyclonal IgG. Antibodies for Akt were obtained from Abcam.

### Statistical analysis

Statistical analyses were performed with SPSS v12.0 (SPSS, Chicago, IL, USA). Data are represented as the mean ± SEM from three independent experiments, unless stated otherwise. Statistical analyses were done using the Student’s *t*-test, and *p* < 0.05 was considered significant.

## Results

### Cell viability after treatment with AE extract

**Fig. 1:**
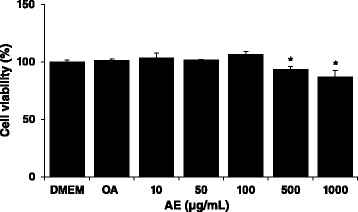
Cell viability effect of *Aralia elata* (Miq) *Seem* (AE) in HepG2 cells. HepG2 cells were treated with oleic aicd (2 mM) and AE extract (10, 50, 100, 500 and 1000 μg/mL). After treatment for 24 h, cell viability was quantified by measuring intracellular levels of ATP. Bars represent the mean ± SEM of 3 experiments done in triplicate

The cytotoxicity of AE in HepG2 cells was determined measuring intracellular level of ATP after incubating cells with AE for 24 h. As shown in Fig. 1, AE exhibited 6.5 % and 13.1 % cytotoxicity in HepG2 cells at concentrations of 500 μg/mL and 1000 μg/mL, respectively. When cells were treated with OA for 24 h to induce conditions of hepatic steatosis, no cytotoxicity was observed in the cells. Therefore, 100 μg/mL of AE and 2.0 mM of OA were used to examine the effect of AE on OA-induced steatosis in HepG2 cell.

### AE decreases lipid accumulation in OA-induced steatotic HepG2 cells

**Fig. 2:**
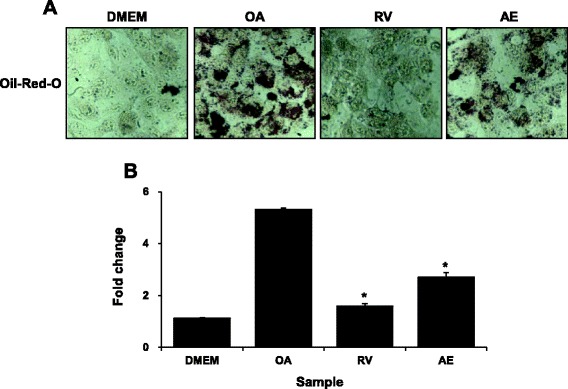
Effects of *Aralia elata* (Miq) *Seem* (AE) on steatosis in HepG2 cells stimulated with oleic acid (OA). **a** HepG2 cells were treated with 100 μg/mL AE. After treatment for 24 h, lipid accumulation was measured by staining with Oil-red-O. **b** Lipid accumulation in HepG2 cell was determined by ORO-based colorimetric assay. Results are the mean ± SEM. *p < 0.05 compared with the OA group. DMEM, control group; OA, oleic acid-treated group; AE, OA + *Aralia elata* (Miq) *Seem*-treated group; RV, OA+ resveratrol-treated group

Incubation of HepG2 cells, for 24 h with 2 mM OA led to increased amounts of intracellular lipid accumulation. Microscopic examination revealed that HepG2 cells treated with OA exhibited significant morphological changes in lipid droplet formation. When cells were treated with AE and OA simultaneously for 24 h, hepatic lipid accumulation significantly decreased (Fig. 2a). There was also a significant decrease in lipid levels by 2.6 folds in AE-treated HepG2 cells (Fig. 2b).

### Changes in expression levels of genes related with lipid metabolism and insulin signaling after AE treatment

**Fig. 3:**
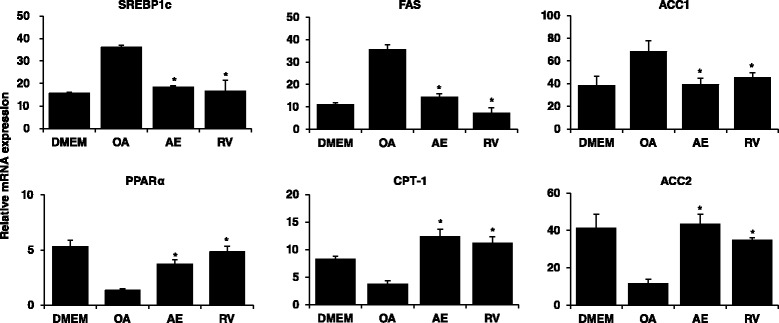
Effects of *Aralia elata* (Miq) *Seem* (AE) on lipid metabolism-associated genes in HepG2 cells. HepG2 cells were treated with 100 μg/mL AE and OA. After treatment for 24 h, RNA was isolated and reverse transcribed for RTPCR analysis using the primers described in materials and methods. Results are the mean ± SEM. *p < 0.05 compared with the OA group. DMEM, control group; OA, oleic acid-treated group; AE, OA+ *Aralia elata* (Miq) *Seem*-treated group; RV, OA+ resveratrol-treated group

Relative mRNA expression levels of lipid metabolism and insulin signaling markers were determined using quantitative PCR. As shown in Fig. 3, mRNA expression levels of hepatic lipogenesis genes such as sterol regulatory element-binding protein 1(SREBP-1), fatty acid synthase(FAS), and acetyl-CoA carboxylase (ACC) 1 increased in the OA-treated cells. AE-treated HepG2 cells showed an inhibition of the mRNA expression levels. Furthermore, mRNA expression levels of peroxisome proliferator activated receptor-α (PPARα), carnitine palmitoyl transferase 1(CPT-1), and ACC2 genes, which are regulators of lipolysis, significantly increased when HepG2 cells were treated with AE. In addition, to demonstrate the effect of AE extract in regulating insulin signaling transduction, we analyzed molecular expression in insulin signaling pathway in HepG2 cells. With AE treatment, the Insulin receptor substrate (IRS) -1/2 mRNA expression significantly increased compared to the OA group. Akt, glucose transport (GLUT2) are the downstream molecules of IRS in insulin pathway. The Akt and GLUT2 expression in OA-treated group was decreased, while AE-treated group increased the hepatic Akt and GLUT2 expression (Additional file 1: Figure S1). These data together suggest strongly an improvement in hepatic lipid metabolism and insulin sensitivity with AE treatment.

### AE increased the glucose consumption

**Fig. 4:**
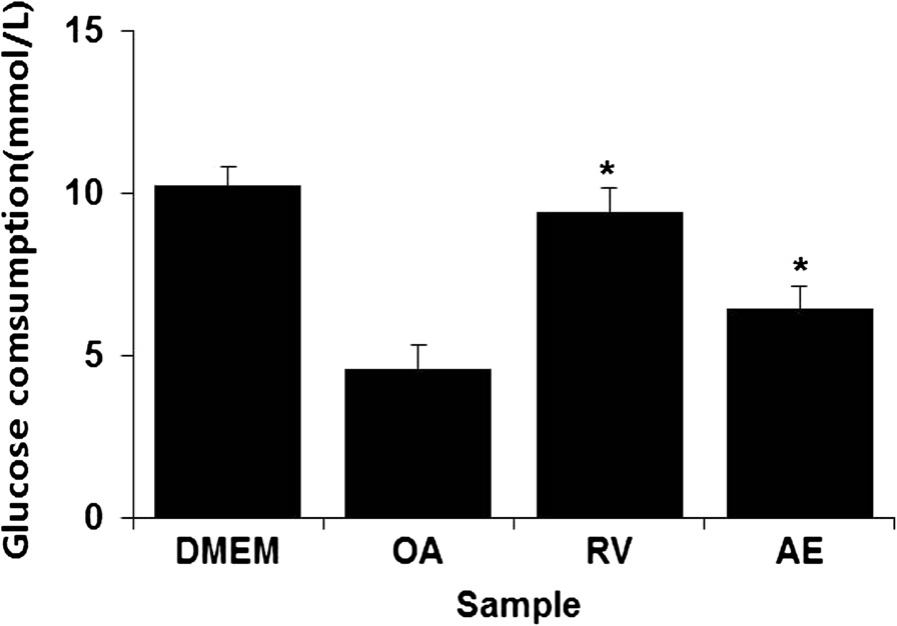
Effects of *Aralia elata* (Miq) *Seem* (AE) on glucose consumption in HepG2 cells. HepG2 cells were treated with 100 μg/mL AE and OA. After treatment for 24 h, glucose consumption was measured using the cell supernatant. Results are the mean ± SEM. *p < 0.05 compared with the OA group. DMEM, control group; OA, oleic acid-treated group; AE, OA+ Aralia elata (Miq) Seem-treated group; RV, OA+ resveratrol-treated group

An excess of glucose, fatty acid, and insulin ultimately leads to hepatic steatosis and worsening of hepatic IR. To determine the effect of AE extract on glucose metabolism and insulin sensitivity, the glucose consumption of OA-induced HepG2 cells was measured. The glucose consumption was decreased in the OA-induced HepG2 cell, while AE extract and RV can significantly enhance the glucose consumption (Fig. 4).

### AE reduces insulin resistance in vivo

**Fig. 5:**
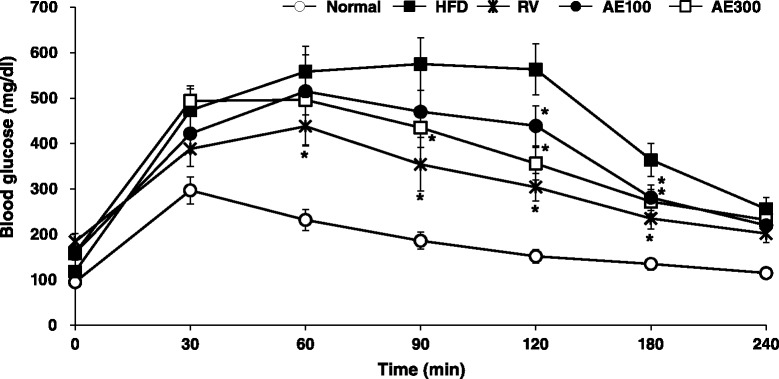
*Aralia elata* (Miq) *Seem* (AE) decreases insulin resistance in OGTT. On the 21th days after AE treatment, OGTT were performed on the animals. Blood samples were collected from tail vein for glucose measurement at 0, 30, 60, 90, 120, 180 and 240 min after glucose administration (po). *p < 0.05 compared with the HFD-fed group. Normal, Normal chaw-fed group; HFD, HFD-fed group; AE100, HFD + *Aralia elata* (Miq) *Seem* 100 mg/kg -treated group; AE300, HFD + *Aralia elata* (Miq) *Seem* 300 mg/kg -treated group; RV, HFD+ resveratrol 300 mg/kg-treated group

To verify whether AE extract can decrease IR in vivo, we performed an animal study using the HFD-induced obese mice. The HFD-fed mice showed significantly higher body weight, serum fasting glucose, insulin levels, and TG levels compared to those observed in the normal group, and HOMA-IR—negatively correlated with insulin sensitivity—also increased in the HFD-fed group. Treatment with AE for 4 weeks resulted in a dose-dependent reduction in body weight and food intake and improved glucose and TG levels. AE extract also significantly decreased HOMA-IR and the serum insulin level, which means that AE reduced IR (Table 2). To supplement these results, the mitigating effect of AE on IR was measured by OGTT. As shown in Fig. 5, mice fed with HFD showed poor glucose tolerance and high fasting blood glucose level. However, the AE-treated groups showed improved glucose tolerance ability and reduced fasting blood glucose level. These results indicate that AE treatment ameliorated IR.

**Table 2:** Basal metabolic parameters

	Normal	HFD	HFD		
			RV	AE100	AE300
Body weight, g	26.1 ± 0.334	42.0 ± 1.766	38.3 ± 0.422^*^	40.4 ± 1.896	37.1 ± 0.712^*^
Food intake, g/day/mouse	3.0 ± 0.114	2.3 ± 0.084	2.03 ± 0.089^*^	2.23 ± 0.119	1.95 ± 0.121^*^
Glucose, mmol/L	8.44 ± 0.25	35.69 ± 1.36	20.36 ± 0.60^*^	32.74 ± 0.99^*^	23.88 ± 1.34^*^
Insulin, μIU/mL	56.3 ± 2.43	489.3 ± 14.3	236.5 ± 6.98^*^	366.5 ± 8.57^*^	215.1 ± 36.2^*^
Triglyceride, mg/dL	0.74 ± 0.012	3.21 ± 0.121	1.21 ± 0.046^*^	2.62 ± 0.088^*^	1.26 ± 0.103^*^
HOMR-IR^a^	0.021	0.776	0.214	0.489	0.228

^*^ *p* < 0.05 compared with the HFD-fed group; ^a^HOMA-IR index was determined as follows: fasting plasma glucose (mmol) × fasting plasma insulin (mU/ml)]/22.5

### AE ameliorates hepatic lipid accumulation

**Fig. 6:**
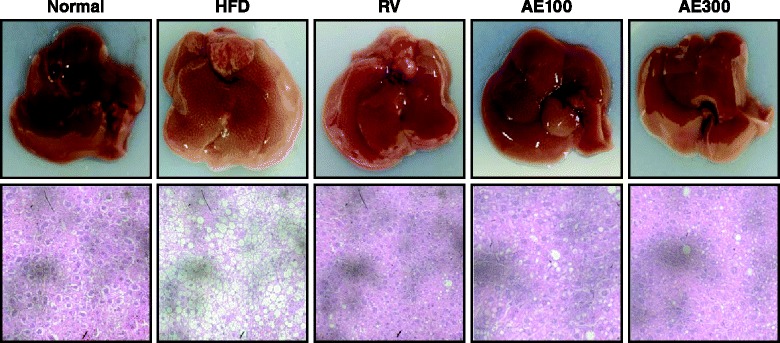
Effect of *Aralia elata* (Miq) *Seem* (AE) extract on hepatic steatosis in HFD-fed mice. Representative microphotograph of hematoxylin and eosin staining of the hepatic lipid accumulation. Normal, Normal chaw-fed group; HFD, HFD-fed group; AE100, HFD + *Aralia elata* (Miq) *Seem* 100 mg/kg -treated group; AE300, HFD + *Aralia elata* (Miq) *Seem* 300 mg/kg -treated group; RV, HFD+ resveratrol 300 mg/kg-treated group

Histological examination of the liver in HFD-fed mice showed lipid accumulation and, eventually, fatty degeneration (ballooning) of hepatocytes (Fig. 6). Treatment with AE extracts at a dose of 300 mg/kg largely attenuated ballooning degeneration, evident by the significant decrease in the formation of fat in the liver sections.

### AE activates the Akt/GLUT4 pathway in vivo

**Fig. 7:**
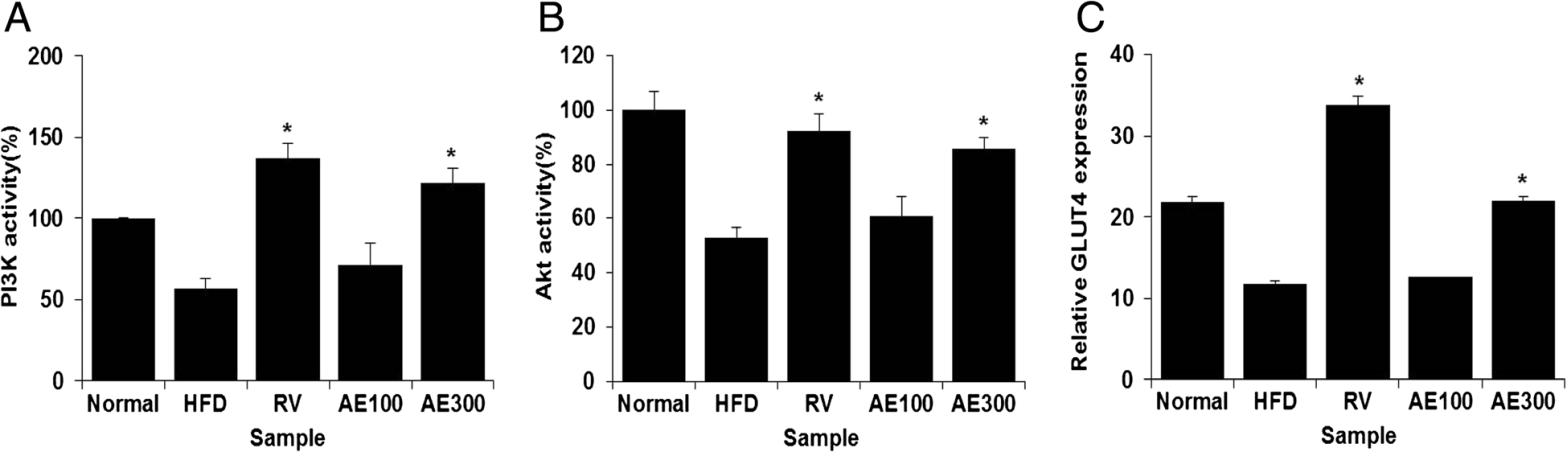
Improved insulin sensitivity in liver and muscle is associated with increased PI3K, Akt2 activities and GLUT4 expression in *Aralia elata* (Miq) *Seem* (AE) extract-treated mice. (A) PI3K activity in liver tissue. (B) Akt2 activity in liver tissue. (C) GLUT4 mRNA expression level in muscle tissue. Results are the mean ± SEM. Results are the mean ± SEM. *p < 0.05 compared with the HFD-fed group. Normal, Normal chaw-fed group; HFD, HFD-fed group; AE100, HFD + *Aralia elata* (Miq) *Seem* 100 mg/kg -treated group; AE300, HFD + *Aralia elata* (Miq) *Seem* 300 mg/kg-treated group; RV, HFD+ resveratrol 300 mg/kg-treated group

Galbo and Shulman [8] reported that feeding rats a high fat diet results in steatosis, increased intra-hepatic DAG content, and impairment of insulin stimulated PI3K signaling. This ultimately leads to the impairment of Akt, which is followed by the translocation of GLUT4. To confirm regulation of PI3 kinase signaling in HFD-fed mice, we measured PI3K and Akt activities in the liver. Figure 7 indicates that PI3K and Akt activities in HFD-fed mice had significantly decreased. Furthermore, we confirmed that PI3K and Akt activities were restored after AE or resveratrol treatment (Fig. 7a and 7b). Additionally, we observed that the expression of GLUT 4 in the skeletal muscles was decreased in OA-treated cells to control cells; treatment with AE or resveratrol significantly elevated the expression of GLUT4 (Fig. 7c). These data suggested that AE may stimulate glucose uptake in skeletal muscles through activation of the Akt/GLUT4 pathway.

## Discussion

NAFLD is characterized by excessive lipid accumulation in the liver in the absence of alcohol consumption and it may progress to NASH, fibrosis, cirrhosis, and hepatocellular carcinoma. Although NAFLD was previously thought to be a benign condition, it is now known to be closely related to the development of IR. Patients with IR have a 4-to11-fold increased risk of developing NAFLD [31, 32].

The association of hepatic steatosis with IR has prompted investigators to elucidate the mechanism underlying NAFLD. The objective of the present study was to elucidate the underlying mechanism of NAFLD by regulating IR. Therefore, the effects of AE on the expression levels of lipogenesis and lipolysis genes and enhancement of the glucose uptake were investigated in OA-induced HepG2 cell. These results indicated that AE extract exerted improvement effect on insulin resistance. And also, we demonstrated the anti-NAFLD effects of AE extract in HFD-fed mice.

We confirmed that AE showed potent anti-NAFLD effects in OA-induced HepG2 cell and HFD-fed mice. Consumption of AE significantly reduced mRNA levels of SREBP1c, FAS and ACC1 and increased PPARα, CPT1 and ACC2. And, administration of AE results in a dose-dependent decrease of food intake and lowering of fasting blood glucose; it also ameliorates hyperinsulinemia. Glucose in OGTT was substantially declined, suggesting the insulin-sensitizing role of AE. In addition, AE extract was significantly attenuated the serum level of TG and lipid accumulation and vacuolar degeneration in the liver. Taken together, AE may decrease lipid accumulation by modulating the expression of key lipid metabolic genes.

Glucose transport is a rate-limiting factor for glucose uptake and metabolism of insulin-sensitive tissue [33]. The glucose transport pathway in skeletal muscles is thought to play an important role in maintaining global glucose homeostasis. The glucose transport in muscle is mainly mediated by GLUT4, which translocates to plasma membrane with the stimulation of insulin. The translocation of GLUT4 is proved to be reduced in IR [34]. The insulin-stimulated translocation of GLUT4 is primarily mediated through Akt/GLUT4 pathway [35]. In this pathway, insulin binds with the insulin receptor leading to the phosphorylation of IRS at multiple tyrosine residues [36]. The activation of IRS results in the phosphorylation of PI3K, which leads to the activation of Akt and subsequently translocation of GLUT4 [37]. In the present study, treatment with AE significantly increased the expression of GLUT4. Elevated GLUT4 expression in AE-treated mice suggested that AE stimulates glucose uptake in skeletal muscles through activation of the GLUT4 pathway. These results showed that Akt/GLUT4 pathway may participate in the regulation of insulin resistance mediated by AE.

Activation of PI3K is a key event in the insulin signaling that leads to the GLUT4 translocation [38]. Akt is another crucial factor in the insulin-regulated glucose transport. Overexpression of constitutively active forms of Akt enhances the glucose transport and GLUT4 translocation without the stimulation of insulin, implicating the important role of Akt in the insulin-stimulated glucose transport [39]. Our results showed that AE extract increased the PI3K and Akt activity, which indicated that AE may improve the insulin resistance in hepatocyte through activating the Akt/GLUT4 pathway.

## Conclusions

The present study indicated that AE exerted improved effect on insulin resistance in HFD-fed mice through activating the Akt/GLUT4 pathway. Our study demonstrated that consumption of AE improved dyslipidemia by through promoting the expression of the lipolytic genes PPARα, ACC2 and CPT1 and inhibiting the expression of lipogenic genes like SREBP-1c, FAS, and ACC1. And AE is also effective in alleviating hyperglycemia and hyperinsulinemia in HFD-fed mice. AE may alleviate IR by through decreasing food intake, reducing intra-abdominal fat deposition, modulating serum levels of IR-related factors, and activating the Akt/GLUT4 pathway. Therefore, our results of this study strongly suggest that administration of AE may be beneficial in retarding the progression of NAFLD or altogether preventing the incidence of the disease.

## Additional file

Additional file 1: Figure S1. Effects of *Aralia elata* (Miq) *Seem* (AE) on insulin signaling-associated genes in HepG2 cells. HepG2 cells were treated with 100 μg/mL AE and OA. After treatment for 24 h, RNA was isolated and reverse transcribed for RTPCR analysis using the primers described in materials and methods. Results are the mean ± SEM. *p < 0.05 compared with the OA group. DMEM, control group; OA, oleic acid-treated group; RV, OA+ resveratrol-treated group; AE, OA+ *Aralia elata* (Miq) *Seem*-treated group. (PPTX 90 kb)

## Abbreviations

NAFLD: Non-alcoholic fatty liver disease
NASH: Non-alcoholic steatohepatitis
IR: Insulin resistance
AE: *Aralia elata* (Miq) *Seem*
DMEM: Dulbecco’s modified Eagle’s medium
FBS: Fetal bovine serum
PS: Penicillin-streptomycin
OA: Oleic acid
DMSO: Dimethyl sulfoxide
PBS: Phosphate-buffered saline
RT-PCR: Reverse transcription polymerase chain reaction
GLUT: Glucose transport
SREBP-1: Sterol regulatory element-binding protein 1
FAS: Fatty acid synthase
ACC: Acetyl-CoA carboxylase
PPARα: Peroxisome proliferator activated receptor-α
CPT-1: Carnitine palmitoyl transferase 1
GAPDH: glyceraldehydes-3-phosphate dehydrogenase
RV: Resveratrol
HFD: High fat diet
ELISA: Enzyme linked immunosorbent assay
TG: Triglycerides
HOMA-IR: Homeostasis model assessment of insulin resistance
OGTT: Oral glucose tolerance test
H&E: Hematoxylin and eosin
PI3K: Phosphoinositide 3 kinase
PKB(Akt): Protein kinase B

## References

[1] Clark JM, Brancati FL, Diehl AM (2002). Nonalcoholic fatty liver disease. Gastroenterology.

[2] Pais R, Ratziu V (2012). Epidemiology and natural history of nonalcoholic fatty liver disease. La Revue du Praticien.

[3] Torres DM, Williams CD, Harrison SA (2012). Features, diagnosis, and treatment of nonalcoholic fatty liver disease. Clin Gastroenterol Hepatol.

[4] Kohjima M, Enjoji M, Higuchi N, Kato M, Kotoh K, Yoshimoto T (2007). Reevaluation of fatty acid metabolism-related gene expression in nonalcoholic fatty liver disease. Int J Mol Med.

[5] Matteoni CA, Younossi ZM, Gramlich T, Boparai N, Liu YC, McCullough AJ (1999). Nonalcoholic fatty liver disease: a spectrum of clinical and pathological severity. Gastroenterology.

[6] Cohen JC, Horton JD, Hobbs HH (2011). Human fatty liver disease: old questions and new insights. Science.

[7] Utzschneider KM, Kahn SE (2006). The role of insulin resistance in nonalcoholic fatty liver disease. J Clin Endocrinol Metab.

[8] Galbo T, Shulman GI (2013). Lipid-induced hepatic insulin resistance. Aging.

[9] Keith GT, Anthony SD (2007). Treatment of non-alcoholic fatty liver disease. J Ther Clin Risk Manage.

[10] Ozturk ZA, Kadayifci A (2014). Insulin sensitizers for the treatment of non-alcoholic fatty liver disease. World J Hepatol.

[11] Stumvoll M, Nurjhan N, Perriello G, Dailey G, Gerich JE (1995). Metabolic effects of metformin in non-insulin-dependent diabetes mellitus. N Engl J Med.

[12] Lin HZ, Yang SQ, Chuckaree C, Kuhajda F, Ronnet G, Diehl AM (2000). Metformin reverses fatty liver disease in obese, leptin deficient mice. Nat Med.

[13] Ahmed MH, Byrne CD (2009). Current treatment of non-alcoholic fatty liver disease. Diabetes Obes Metab.

[14] Hwang YJ, Wi HR, Kim HR, Park KW, Hwang KA (2014). Pinus densiflora Sieb. et Zucc. Alleviates Lipogenesis and Oxidative Stress during Oleic Acid-Induced Steatosis in HepG2 Cells. Nutrients.

[15] Kang JS, Lee WK, Lee CW, Yoon WK, Kim N, Park SK (2011). Improvement of high-fat diet-induced obesity by a mixture of red grape extract, soy isoflavone and L-carnitine: Implications in cardiovascular and non-alcoholic fatty liver diseases. Food Chem Toxicol.

[16] Yun JW (2010). Possible anti-obesity therapeutics from nature – a review. Phytochemistry.

[17] Anhê FF, Roy D, Pilon G, Dudonné S, Matamoros S, Varin TV, et al. A polyphenol-rich cranberry extract protects from diet-induced obesity, insulin resistance and intestinal inflammation in association with increased Akkermansia spp. population in the gut microbiota of mice. Gut. 2014, doi: 10.1136/gutjnl-2014-307142. 10.1136/gutjnl-2014-307142 25080446

[18] Chiu WC, Yang HH, Chiang SC, Chou YX, Yang HT. Auricularia polytricha aqueous extract supplementation decreases hepatic lipid accumulation and improves antioxidative status in animal model of nonalcoholic fatty liver. Biomedicine (Taipei). 2014. doi: 10.7603/s40681-014-0012-3 10.7603/s40681-014-0012-3 PMC4265006 25520925

[19] Xinghai Z, Ying G, Jinwei X, Xiaohui L, Feng J, Bo L (2014). Inhibitory Effect of Tea (Camellia Sinensis (L.) O. Kuntze, Theaceae) Flower Extracts on Oleic Acid-Induced Hepatic Steatosis in Hepg2 Cells. J Food Nutr Res.

[20] Wan Y, Liu LY, Hong ZF, Peng J (2014). Ethanol extract of Cirsium japonicum attenuates hepatic lipid accumulation via AMPK activation in human HepG2 cells. Exp Ther Med.

[21] Li M, Lu W (2009). Pharmacological research progress of Araliaelata. Medical Recapitulate.

[22] Lee JH, Jeong CS, Lee JH, Jeong CS (2009). Suppressive effects on the biosynthesis of inflammatory mediators by Aralia elata extract fractions in macrophage cells. Environ Toxicol Pharmacol.

[23] Nhiem NX, Lim HY, Kiem PV, Minh CV, Thu VK, Tai BH (2011). Oleanane-type triterpene saponins from the bark of Aralia elata and their NF-κB inhibition and PPAR activation signal pathway. Bioorg Med Chem Lett.

[24] Tomatsu M, Kameyama M, Shibamoto NA (2003). Aralin, A new cytotoxic protein from Aralia elata, inducing apoptosis in human cancer cells. Cancer Lett.

[25] Suh SJ, Jin UH, Kim KW (2007). Triterpenoid saponin, oleanolic acid 3-O-d-glucopyranosyl(1 → 3)-alpha-l-rhamnopyranosyl(1 → 2)-alpha-l arabinopyranoside (OA) from Aralia elata inhibits LPS-induced nitric oxide production by down regulated NF-kappa B in raw 264.7 cells. Arch Biochem Biophys.

[26] Shin KH, Cho SY, Lee MK, Lee JS, Kim MJ (2004). Effects of Aralia elata, Acanthopanacis cortex and Ulmus davidiana Water Extracts on Plasma Biomarkers in Streptozotocin - Induced Diabetic Rats. J Korean Soc Food Sci Nutr.

[27] Kim YH, Im JG (1999). Effect of saponin from the shoot of Aralia elata in normal rats and streptozotocin induced diabetic rats. J Korean Soc Food Sci Nutr.

[28] Cousin SP, Hügl SR, Wrede CE, Kajio H, Myers MG, Rhodes CJ (2001). Free fatty acid-induced inhibition of glucose and insulin-like growth factor I-Induced deoxyribonucleic acid synthesis in the pancreatic beta-Cell Line INS-1. Endocrinology.

[29] Matthews DR, Hosker JP, Rudenski AS, Naylor BA, Treacher DF, Turner RC (1985). Homeostasis model assessment: insulin resistance and beta-cell function from fasting plasma glucose and insulin concentrations in man. Diabetologia.

[30] Samuel VT, Liu ZX, Qu X, Elder BD, Bilz S, Befroy D (2004). Mechanism of hepatic insulin resistance in non-alcoholic fatty liver disease. J Biol Chem.

[31] Nagle CA, Klett EL, Coleman RA (2009). Hepatic triacylglycerol accumulation and insulin resistance. J Lipid Res.

[32] Ruhl CE, Everhart JE (2004). Epidemiology of nonalcoholic fatty liver. Clin Liver Dis.

[33] Liu M, Wu K, Mao X, Wu Y, Ouyang J (2010). Astragalus polysaccharide improves insulin sensitivity in KKAy mice: regulation of PKB/GLUT4 signaling in skeletal muscle. J Ethnopharmacol.

[34] Minokoshi Y, Kahn CR, Kahn BB (2003). Tissue-specific Ablation of the GLUT4 Glucose Transporter or the Insulin Receptor Challenges Assumptions about Insulin Action and Glucose Homeostasis. J Biol Chem.

[35] Samuel VT, Shulman GI (2012). Mechanisms for insulin resistance: common threads and missing links. Cell.

[36] Wang X, Wahl R (2014). Responses of the insulin signaling pathway in the brown adipose tissue of rats following cold exposure. PLoS One.

[37] Hsieh TJ, Hsieh PC, Wu MT, Chang WC, Hsiao PJ, Lin KD (2011). Betel nut extract and arecoline block insulin signaling and lipid storage in 3 T3-L1 adipocytes. Cell Biol Toxicol.

[38] Choi SS, Cha BY, Iida K, Lee YS, Yonezawa T, Teruya T (2011). Artepillin C, as a PPARγ ligand, enhances adipocyte differentiation and glucose uptake in 3 T3-L1 cells. Biochem Pharmacol.

[39] Foran PG, Fletcher LM, Oatey PB, Mohammed N, Dolly JO, Tavare JM (1999). Protein kinase B stimulates the translocation of GLUT4 but not GLUT1 or transferrin receptors in 3 T3-L1 adipocytes by a pathway involving SNAP-23, Synaptobrevin-2, and/or cellubrevin. J Biol Chem.

