# Supplementary material for: Extracts from Aralia elata (Miq) Seem alleviate hepatosteatosis via improving hepatic insulin sensitivity
Source: BMC Complement Altern Med. 2015 Oct 5;15:347. doi: 10.1186/s12906-015-0871-5 (PMC4595215; doi:10.1186/s12906-015-0871-5)
Supplement: Additional file 1: Figure S1. — Effects of Aralia elata (Miq) Seem (AE) on insulin signaling-associated genes in HepG2 cells. HepG2 cells were treated with 100 μg/mL AE and OA. After treatment for 24 h, RNA was isolated and reverse transcribed for RTPCR analysis using the primers described in materials and methods. Results are the mean ± SEM. *p < 0.05 compared with the OA group. DMEM, control group; OA, oleic acid-treated group; RV, OA+ resveratrol-treated group; AE, OA+ Aralia elata (Miq) Seem-treated group. (PPTX 90 kb) [file 12906_2015_871_MOESM1_ESM.pptx]

## Slide 1
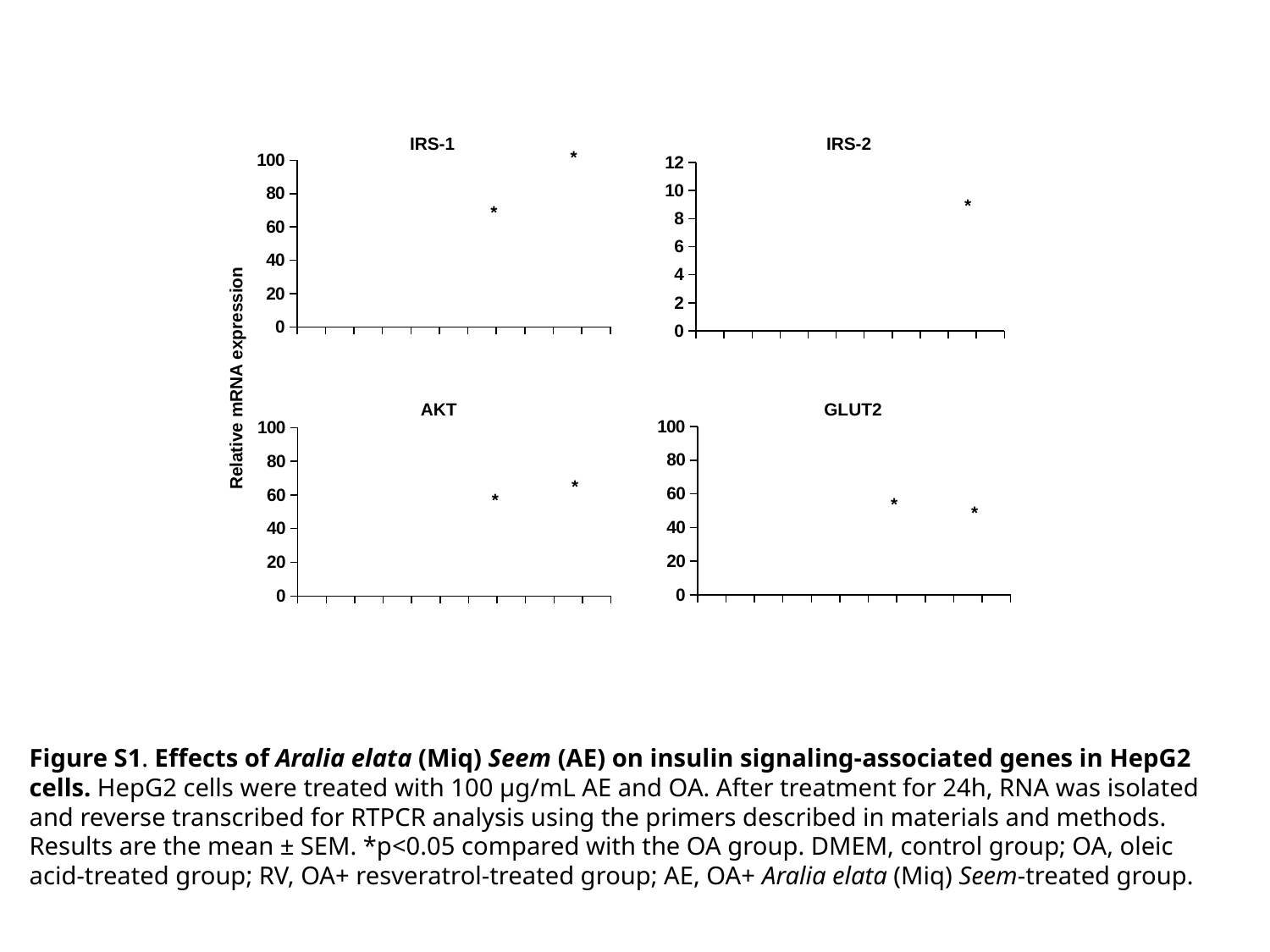

IRS-1
IRS-2
*
### Chart
| Category | |
|---|---|
### Chart
| Category | |
|---|---|*
*
Relative mRNA expression
AKT
GLUT2
### Chart
| Category | |
|---|---|
### Chart
| Category | |
|---|---|*
*
*
*
Figure S1. Effects of Aralia elata (Miq) Seem (AE) on insulin signaling-associated genes in HepG2 cells. HepG2 cells were treated with 100 μg/mL AE and OA. After treatment for 24h, RNA was isolated and reverse transcribed for RTPCR analysis using the primers described in materials and methods. Results are the mean ± SEM. *p<0.05 compared with the OA group. DMEM, control group; OA, oleic acid-treated group; RV, OA+ resveratrol-treated group; AE, OA+ Aralia elata (Miq) Seem-treated group.
